# Atmosphere-Driven Phase Engineering in MoO_*x*_: From α‑MoO_3_ Microbelts to MoO_2_ Revealed by Atomic-Scale Hyperfine Probes

**DOI:** 10.1021/acsmaterialsau.6c00153

**Published:** 2026-10-15

**Authors:** Katiusse Soares de Souza, Crystian Willian C. Silva, Gabriel A. Cabrera-Pasca, Juliana Schell, Artur Wilson Carbonari

**Affiliations:** † Instituto de Pesquisas Energéticas e Nucleares, IPEN-CNEN/SP, São Paulo 05508-000, Brazil; ‡ Universidade Federal do Pará (UFPA), Abaetetuba, Pará 09210-580, Brazil; § 30531 European Organization for Nuclear Research (CERN), Geneva CH-1211, Switzerland; ∥ Institute for Materials Science and Center for Nanointegration Duisburg-Essen (CENIDE), University of Duisburg-Essen, Essen 45141, Germany

**Keywords:** molybdenum oxides, phase transitions, spectroscopy, hyperfine interactions, local structure

## Abstract

This work reports a comprehensive investigation of the structural, morphological, and local-scale evolution of molybdenum oxides (MoO_*x*_) derived from a chelated precursor obtained via thermal decomposition of molybdenum­(II) acetate. By systematically controlling annealing temperature and atmosphere, we demonstrate a clear pathway for phase and morphology engineering across the Mo–O system. Under oxidizing conditions, α-MoO_3_ microbelts are stabilized up to 800 °C and undergo a morphology-driven transformation into nanosheets without phase alteration. In contrast, vacuum annealing induces reduction processes, leading to the formation of β-MoO_3_ and its subsequent irreversible transformation into MoO_2_. The β-MoO_3_ → MoO_2_ transition is probed at the atomic scale using time-differential perturbed angular correlation (TDPAC) spectroscopy with the ^111^In­(^111^Cd) probe nucleus over the temperature range 25–600 °C. The evolution of the hyperfine parameters reveals two distinct regimes associated with progressive local ordering and with the emergence of a likely associated with probable MoO_2_-related environment, characterized by a quadrupole frequency of ∼149 MHz. Correlation analysis and hierarchical clustering provide quantitative evidence of an irreversible transition above 600 °C. Structural characterization after TDPAC measurements as a function of temperature confirms the predominance of monoclinic MoO_2_ (∼90%) with residual β-MoO_3_, establishing a direct link between local hyperfine signatures and macroscopic phase composition. These results highlight the critical role of atmosphere in dictating phase transformation pathways and demonstrate that TDPAC is a powerful tool for resolving early stage local structural evolution. The findings provide a robust framework for the rational design of MoO_*x*_-based materials with tunable structural, electronic, and catalytic properties.

## Graphical abstract

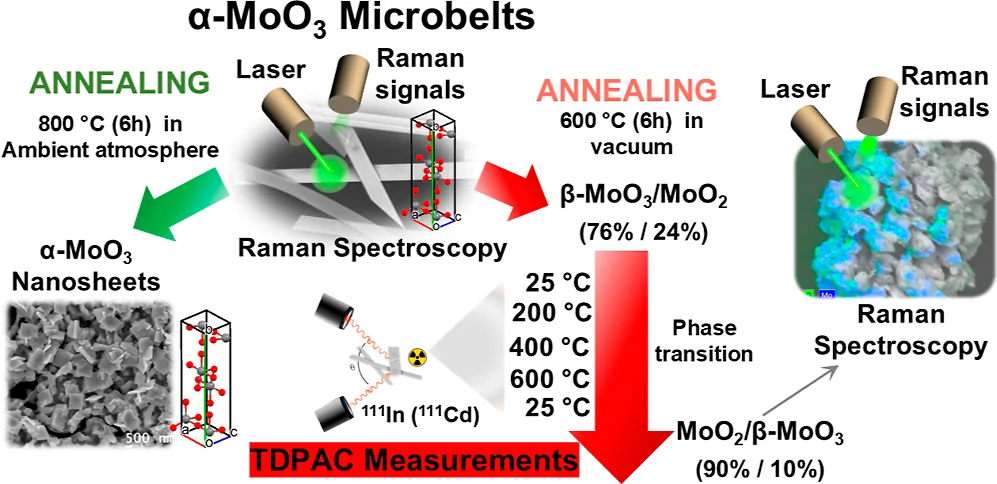

## Introduction

Transition-metal oxides at the micro- and nanoscale constitute a central class of materials for advanced technological applications, owing to their rich interplay between structure, morphology, and electronic properties. In particular, molybdenum oxides (MoO_*x*_) have emerged as highly versatile systems, exhibiting adjustable optical, catalytic, and electronic functionalities that support applications in sensing, energy storage, medical therapy, and nuclear medicine.^1–3^

Among the various stoichiometries, molybdenum trioxide (MoO_3_) and molybdenum dioxide (MoO_2_) represent two limiting oxidation states with markedly distinct physical properties. While the thermodynamically stable α-MoO_3_ (orthorhombic, with *a* = 3.96 Å, *b* = 13.85 Å, *c* = 3.69 Å) is a layered, wide-bandgap semiconductor with pronounced anisotropy,^4–6^ MoO_2_ (monoclinic, *a* = 5.625 Å, *b* = 4.872 Å, *c* = 5.645 Å) exhibits metallic-like conductivity and enhanced catalytic activity.^7^ The existence of multiple polymorphs of MoO_3_ (α, β, and *h* phases) further enriches the phase landscape, making the Mo–O system particularly sensitive to synthesis conditions and thermodynamic parameters. This sensitivity provides opportunities for phase engineering, but also introduces challenges in achieving controlled and reproducible synthesis. Although generally considered weakly paramagnetic, Mo or O vacancies can induce ferromagnetically coupled magnetic moments, and diffraction studies evidence a structural transition near 540 °C and an electronic/magnetic transition around 493 °C.^8–10^

A key aspect in tailoring MoO_*x*_ materials lies in understanding and controlling phase transitions, especially those driven by temperature and oxygen partial pressure. While the α → β transformation and the reduction toward MoO_2_ have been reported, the microscopic mechanisms governing these transitions (particularly at the local atomic scale) remain insufficiently understood. Most studies rely on bulk-sensitive techniques, which often obscure the early stage evolution of local environments that ultimately dictate phase stability and transformation pathways.

From a synthesis perspective, chemical routes such as sol–gel and coprecipitation methods frequently employ precursors such as molybdates (sodium or ammonium), which predominantly result in the formation of molybdenum sulfides and oxides.^11–13^ Thermal decomposition routes offer a versatile and scalable approach for producing MoO_*x*_ nanostructures, enabling control over nucleation and growth processes through ligand chemistry and reaction conditions, providing, therefore significant control over morphology and structure.^14,15^ In this method, a metal–organic precursor is decomposed in a high-boiling-point solvent in the presence of ligands that adsorb onto nascent nuclei, reduce surface free energy, and thereby regulate particle size and shape.^15–17^ Oleylamine and oleic acid are the most reported ligand pair: oleylamine acts as a mild reducing agent (decomposition temperature ≃ 240 °C), while oleic acid stabilizes particle growth and provides an organic surface coating.^18–20^ Palm kernel oil, extracted from *Elaeis guineensis* and rich in saturated and unsaturated fatty acids, has recently emerged as a green alternative ligand in thermal decomposition synthesis.^14,19^

The literature frequently reports the use of acetylacetonates for producing transition-metal nanostructures (Ni, Co, Cu).^21–23^ For molybdenum specifically, the most common precursors are molybdenum hexacarbonyl and ammonium molybdate.^24–26^ Molybdenum­(II) acetate is a less explored but promising candidate: it is a binuclear complex featuring a quadruple Mo–Mo bond (σ + π + 2δ) with an intermetallic distance of only 2.11 Å and temperature-dependent para- to diamagnetic behavior.^27,28^ However, synthesizing MoO_*x*_ nanostructures from this precursor via chemical routes presents challenges, including the need for strong acids, low product crystallinity, and broad size distributions. Subtle variations in precursor type, pH, atmosphere, or ligand can trigger phase changes, and postsynthesis thermal treatments (500–800 °C) used to improve crystallinity may simultaneously generate defects, vacancies, and undesired phase transitions.^29–32^

The use of unconventional ligands and precursors, such as molybdenum­(II) acetate combined with organic coordinating media, introduces additional degrees of freedom in directing structural evolution. However, such systems often yield complex intermediate states (e.g., chelated or amorphous phases), whose transformation into crystalline oxides is highly sensitive to postsynthesis treatments.

To address these challenges, local-probe techniques capable of investigating atomic-scale environments are essential. Time-Differential Perturbed Angular Correlation (TDPAC) spectroscopy, based on a nuclear quantum effect, enables investigation of the local atomic environment of probe nuclei through the hyperfine interactions between their nuclear moments and the electric and/or magnetic fields present in their vicinity. In the case of the electric quadrupole interaction, the nuclear quadrupole moment interacts with the electric field gradient (EFG) at the probe site, whose magnitude and symmetry reflect the local electronic distribution and the configuration of the surrounding atoms. This interaction produces a time-dependent modulation of the angular correlation between the γ-rays emitted in cascade, from which hyperfine parameters, such as the quadrupole frequency and the asymmetry parameter, can be determined. These parameters make it possible to distinguish different local environments occupied by the probe nuclei and to track changes in local ordering and symmetry associated with structural modifications and phase transformations.^14,23,33–36^

In this work, we investigate the formation and transformation of MoO_*x*_ micro- and nanostructures derived from a chelated precursor obtained via thermal decomposition of molybdenum­(II) acetate in the presence of oleylamine and palm kernel oil. By combining conventional structural and morphological characterization with temperature-dependent TDPAC measurements using the ^111^In­(^111^Cd) probe, we establish a direct correlation between macroscopic phase evolution and local hyperfine interactions. This research study presents a graphical summary of the methods and findings in Figure 1, showcasing the methodology and results of the study.

**Figure 1:**
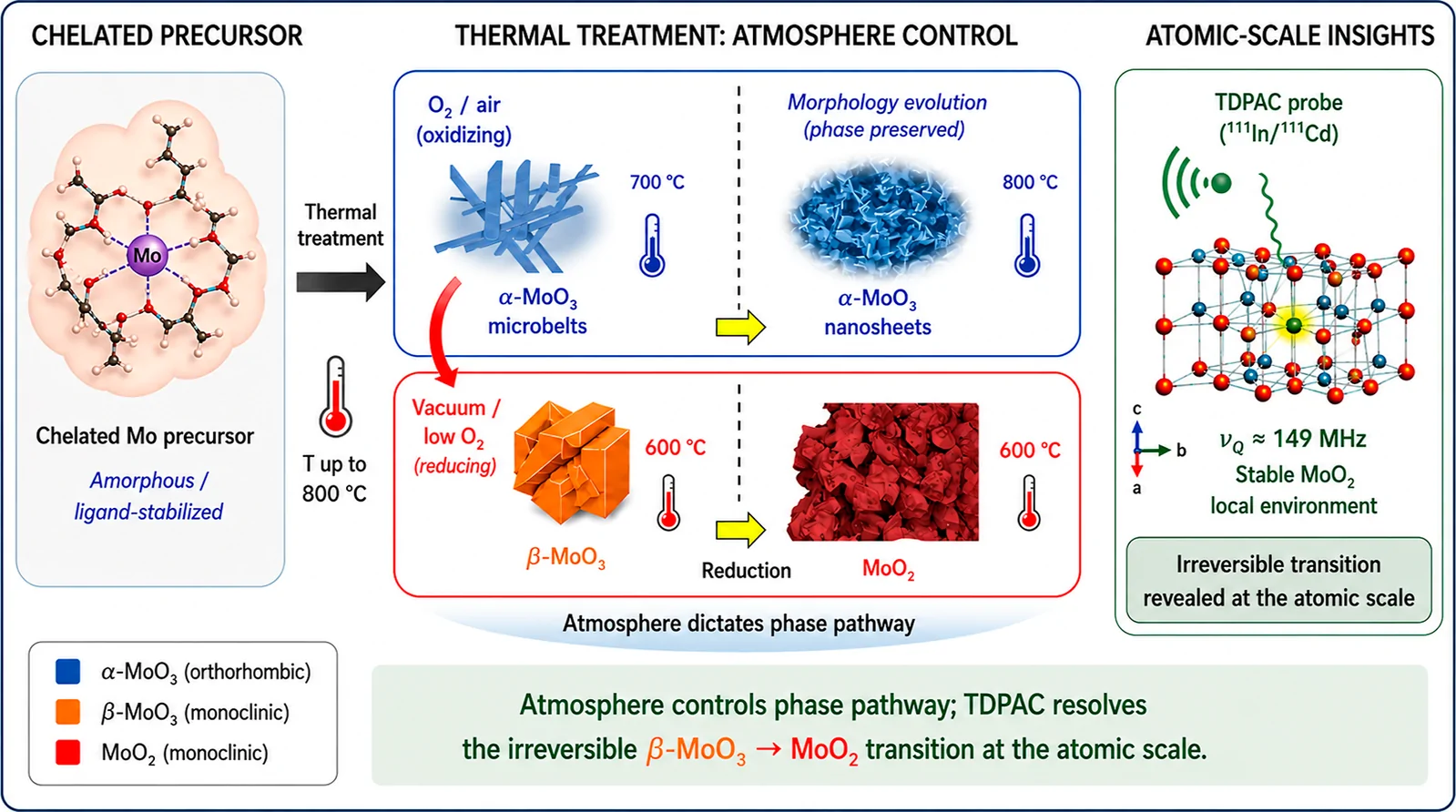
Scheme illustrating the methodology and key findings of the research presented in this report.

## Results and Discussion

In the context of using molybdenum­(II) acetate mediated by the oleylamine/palm kernel oil ligand pair as an alternative route for synthesizing molybdenum nanostructures, the results suggest that the adopted methodology resulted in the formation of a complex material or chelate based on molybdenum. These complexes are formed by metal ions that are bound or coordinated to organic compounds.^37^

In the employed methodology, the formation of the complex material (chelate) is a result of the interaction between the binuclear precursor and the ligand pair (oleylamine/palm kernel oil). Oleylamine acts as a reducing agent, while the fatty acids present in palm kernel oil serve as stabilizing agents.^14^ However, due to the varying chain lengths (capric acid, lauric acid, myristic acid, palmitic acid, oleic acid, and linoleic acid) and degrees of saturation, palm kernel oil leads to a heterogeneous stabilization process. This, combined with the intrinsic characteristics of molybdenum­(II) acetate, promotes the formation of a complex or chelate.

Molybdenum exhibits the electronic configuration [K]­4d^5^5s^1^, which implies that these electrons have lower binding energy to the molybdenum atom than their counterparts in other transition metals. This results in the formation of molecular orbitals with lower-energy charge-transfer states, a characteristic that favors complex formation in the presence of amine and carboxyl groups.^38^

This hypothesis is supported by the results obtained from FTIR, XRD, and SEM analyses for this sample. Figure 2 presents the structural and morphological characterization results obtained for this sample. Figure 2a shows the FTIR spectrum, in which bands located between 721 cm^–1^ and 996 cm^–1^ are observed, corresponding to metal–oxygen (M–O) bonds, which are typical of oxide materials. The bands at 2919 cm^–1^ and 2858 cm^–1^ correspond to the asymmetric and symmetric stretching vibrations of CH_2_, respectively. These bands are common to oleyl groups, composed of saturated and unsaturated fatty acids, and can be attributed to palm kernel oil.^39^ The region around 1375 cm^–1^ corresponds to the OH deformation mode, while the bands between 3332 cm^–1^ and 3297 cm^–1^ are associated with NH stretching vibrations. Bands related to carboxyl groups coordinated to transition metals appear in the range of approximately 1600 cm^–1^ to 1650 cm^–1^.^40,41^

**Figure 2:**
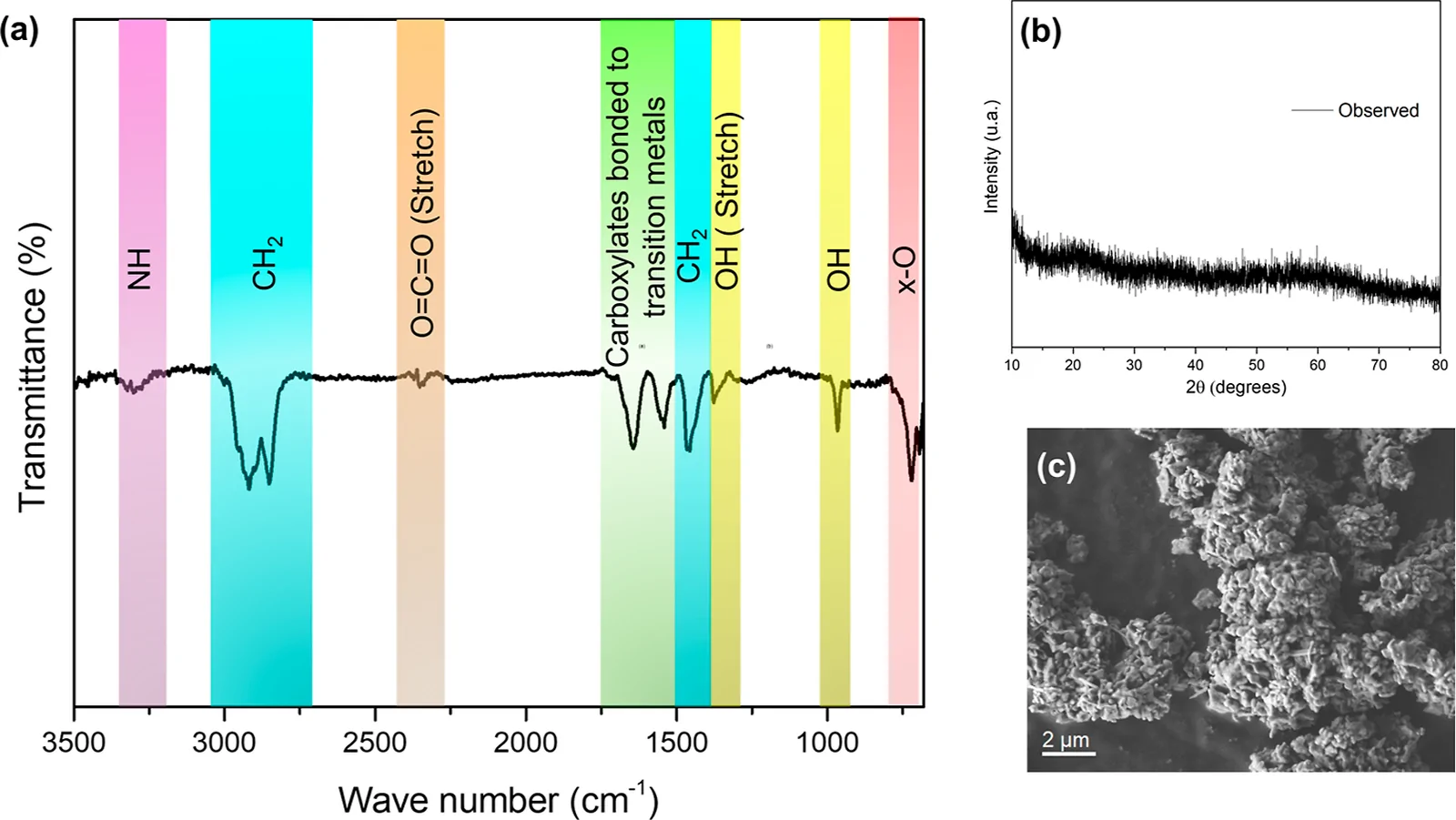
Results of the chemical, morphological, and structural characterization of the synthesized product (chelate): (a) FTIR spectrum highlighting the observed vibrational bands; (b) X-ray diffraction pattern characteristic of amorphous materials; (c) scanning electron microscopy image of the sample, showing a surface with no predominant well-defined morphology.

The FTIR results indicate the presence of Mo bonded to carboxyl groups, suggesting that the organometallic precursor used (molybdenum­(II) acetate) was reduced and stabilized by the ligands. However, there are still Mo–O bonds present, indicating that the nucleation and growth processes occurred heterogeneously, which facilitated the reassociation of Mo and O atoms.^40^

From a different perspective, the X-ray diffraction pattern (see Figure 2b) of the chelate film deposited on a glass substrate displays characteristics commonly seen in amorphous materials. One notable feature is the lack of distinct peaks, as well as generally low intensity counts.^42^ In terms of morphology, the scanning electron microscopy images reveal the presence of a solid with an undefined shape, covered by organic material (see Figure 2c).

### Annealing for the Crystallization of MoO_*x*_-Based Systems

In the process of obtaining molybdenum oxides from molybdenum­(II) acetate, previous studies have shown that annealing is an effective method for the crystallization of these systems. The literature suggests that factors such as temperature and atmosphere play a crucial role in determining the structural and morphological properties of the resulting material.^40^

In this study, the chelate synthesized was annealed at 700 °C for two different durations (10 min and 3 h) under an ambient (oxidizing) atmosphere. The results of the morphological and structural analysis after 10 min, the absence of well-defined diffraction peaks indicates that, under the experimental conditions employed, the short-duration heat treatment does not induce long-range crystallographic order detectable by XRD. Although SEM micrographs reveal the formation of relatively large, heterogeneous agglomerated structures, this morphological evolution does not necessarily imply the development of crystallinity. The observed changes may be associated with processes of structural relaxation, local reorganization, and the coalescence or aggregation of the material, which remains predominantly amorphous. Therefore, the results suggest that morphological reorganization can occur without the simultaneous establishment of long-range crystalline order detectable by X-ray diffraction (see Supporting Information, Figure S1).^43^

However, samples annealed for 3 h show the formation of well-defined microbelts with crystalline structure, as shown in Figure 3. The images in Figure 3a–h depict the morphological characterization of this sample, revealing the presence of molybdenum oxide microbelts with an average length of 37 μm (*L*_*m*_ = 37 μm) and a standard deviation of 2 μm (σ = ± 2 μm). The average thickness of the microbelts is 744 nm (*T*_*m*_ = 744 nm), with a standard deviation of 297 nm (σ = ± 297 nm), both values obtained through Gaussian fitting.

**Figure 3:**
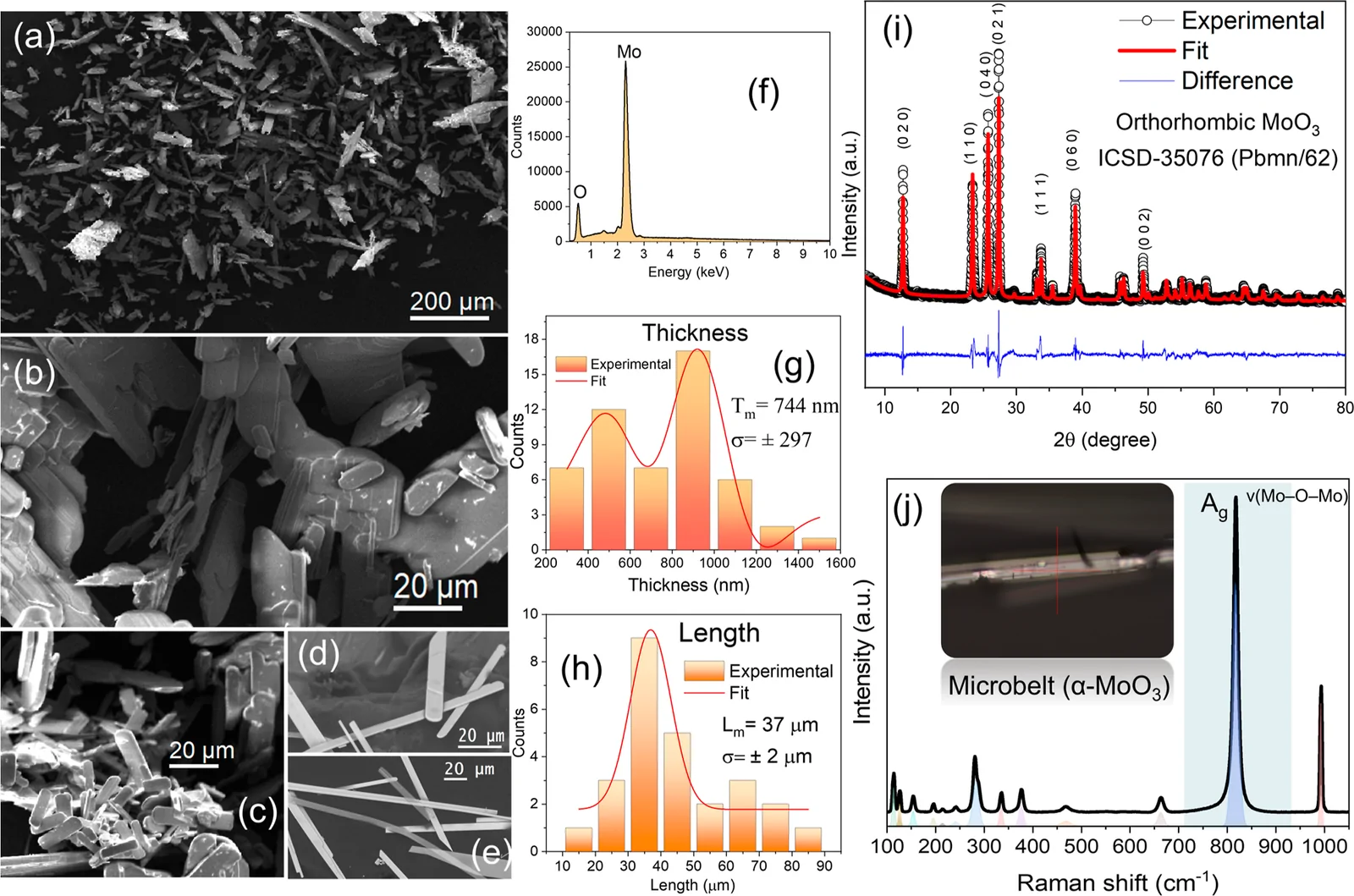
Results of XRD, SEM/EDS, and Raman analyses of the sample subjected to annealing at 700 °C for 3 h. (a–e) Scanning electron microscopy (SEM) micrographs acquired at different magnifications, evidencing a well-defined microbelt morphology; (d,e) surface regions of the sample; (f) EDS analysis revealing only Mo and O atoms; (g) thickness distribution of the sample; (h) width distribution of the sample; (i) X-ray diffraction pattern confirming the α-MoO_3_ structure; and (j) Raman spectrum highlighting the characteristic bands of α-MoO_3_.

The X-ray diffraction results shown in Figure 3i confirm the crystalline structure of these microbelts. The diffraction pattern obtained corresponds to the orthorhombic space group *Pbnm*/62 (ICSD-35076) of molybdenum trioxide (α-MoO_3_). The experimental pattern also indicates a preferred orientation along the (021) plane, suggesting an anisotropic character that is consistent with the observed morphology. This anisotropic nature is attributed to the intrinsically anisotropic structure of α-MoO_3_, where layers of MoO_6_ units are connected by van der Waals interactions. This favors the growth of two-dimensional structures such as belts, sheets, and needles, as reported in previous studies.^44,45^

The Raman spectroscopy results presented in Figure 3j further support the anisotropic nature of the microbelts. The Raman spectrum shows a well-defined set of bands that are in excellent agreement with the assignments reported in the literature for the orthorhombic α-MoO_3_ phase. For example, the most intense band observed at 817 cm^–1^ is assigned to the *A*_*g*_ vibrational mode (ν­(Mo–O–Mo) stretching), which involves bridging bonds between MoO_6_ octahedra and is characteristic of the layered structure of α-MoO_3_.^46^ Similarly, the band at 993 cm^–1^ is attributed to a Raman-active mode of *A*_*g*_ symmetry, which is associated with the terminal ν­(Mo=O) stretching vibration and is considered the main vibrational fingerprint of α-MoO_3_.^46^

In the intermediate spectral region, the band at 664 cm^–1^ is assigned to a *B*_3*g*_ symmetry mode associated with Mo–O–Mo network stretching vibrations, while the peak at 469 cm^–1^ (an *A*_*g*_ mode) is related to bending and stretching vibrations of the MoO_6_ octahedral units. The bands observed at 376 cm^–1^ (*B*_1*g*_) and 335 cm^–1^ (*A*_*g*_) correspond to bending and internal Mo–O stretching modes, which are typical of the α-MoO_3_ crystalline lattice.^47^ The low-frequency bands, located between 280 and 110 cm^–1^, are predominantly attributed to lattice modes involving collective translational and rotational motions of the MoO_6_ octahedra.^46,47^

### Influence of Temperature and Atmosphere on the Structural and Morphological Properties of α-MoO_3_ Microbelts

In this study, we investigated the influence of temperature and atmosphere on the structural and morphological properties of α-MoO_3_ microbelts. The microbelts were divided into two groups and subjected to different thermal treatments: one group was treated at 800 °C in an oxygen-rich atmosphere, while the other was treated at 600 °C under vacuum.

The results for the microbelts treated at 800 °C in an ambient atmosphere for 6 h are presented in Figure 4. The X-ray diffraction pattern in Figure 4a shows that the microbelts maintained their orthorhombic structure, but there was a decrease in the preferential crystallographic orientation compared to the untreated sample (Figure 3).

**Figure 4:**
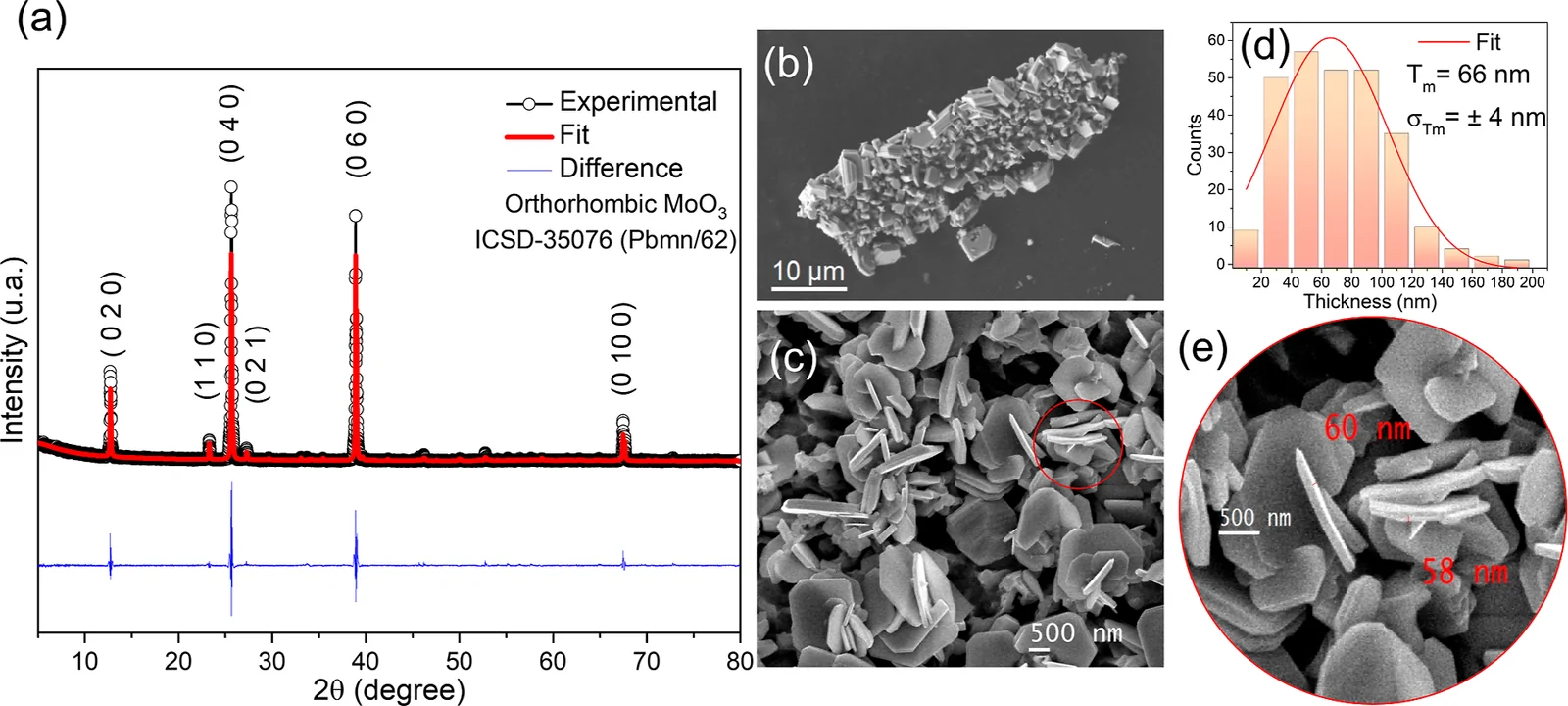
Structural and morphological results for the sample thermally treated at 800 °C for 6 h under an oxidizing atmosphere: (a) X-ray diffraction pattern evidencing the α-MoO_3_ phase; (b–e) scanning electron microscopy analysis; (b) degraded region of the microbelts; (c) surface region of the sample highlighting the nanosheet morphology; (d) thickness distribution; (e) magnified view of (c), highlighting the nanosheet thickness.

Scanning electron microscopy (SEM) images in Figure 4b–e reveal significant changes in the morphology of the microbelts after thermal treatment. The microbelts were covered with α-MoO_3_ nanosheets, with an average thickness of 66 nm (*E*_*m*_ = 66 nm) and a standard deviation of 4 nm (σ*E*_*m*_ = ± 4 nm), as determined by a Gaussian fit to the thickness distribution. This suggests that the nanosheets were formed through a vapor–solid growth mechanism, with the microbelts acting as a substrate. Figure 4b shows a partially degraded microbelt with a high density of nanosheets on its surface, indicating that the nanosheets grew on the microbelt.

This resulted in the loss of preferential crystallographic orientation at the macroscopic scale, as the secondary growth occurred in a nonepitaxial manner with randomly distributed orientations. However, at the local scale, each nanosheet exhibited anisotropic growth, consistent with the lamellar structure of α-MoO_3_. Despite the morphological changes, the α-MoO_3_ crystalline phase was preserved due to the high availability of oxygen in the processing atmosphere. This is in agreement with previous studies in the literature.^48,49^

The samples thermally treated at 600 °C for 6 h under vacuum atmosphere were characterized by X-ray diffraction (XRD) in order to investigate possible structural modifications induced by the thermal treatment. Figure 5 shows the obtained XRD pattern, whose refinement using the Rietveld method evidence the coexistence of two crystalline phases: the β-MoO_3_ phase (ICSD-644067) as the major phase (approximate contribution of 76%) and the monoclinic MoO_2_ phase as a secondary phase, with an approximate contribution of 24%.

**Figure 5:**
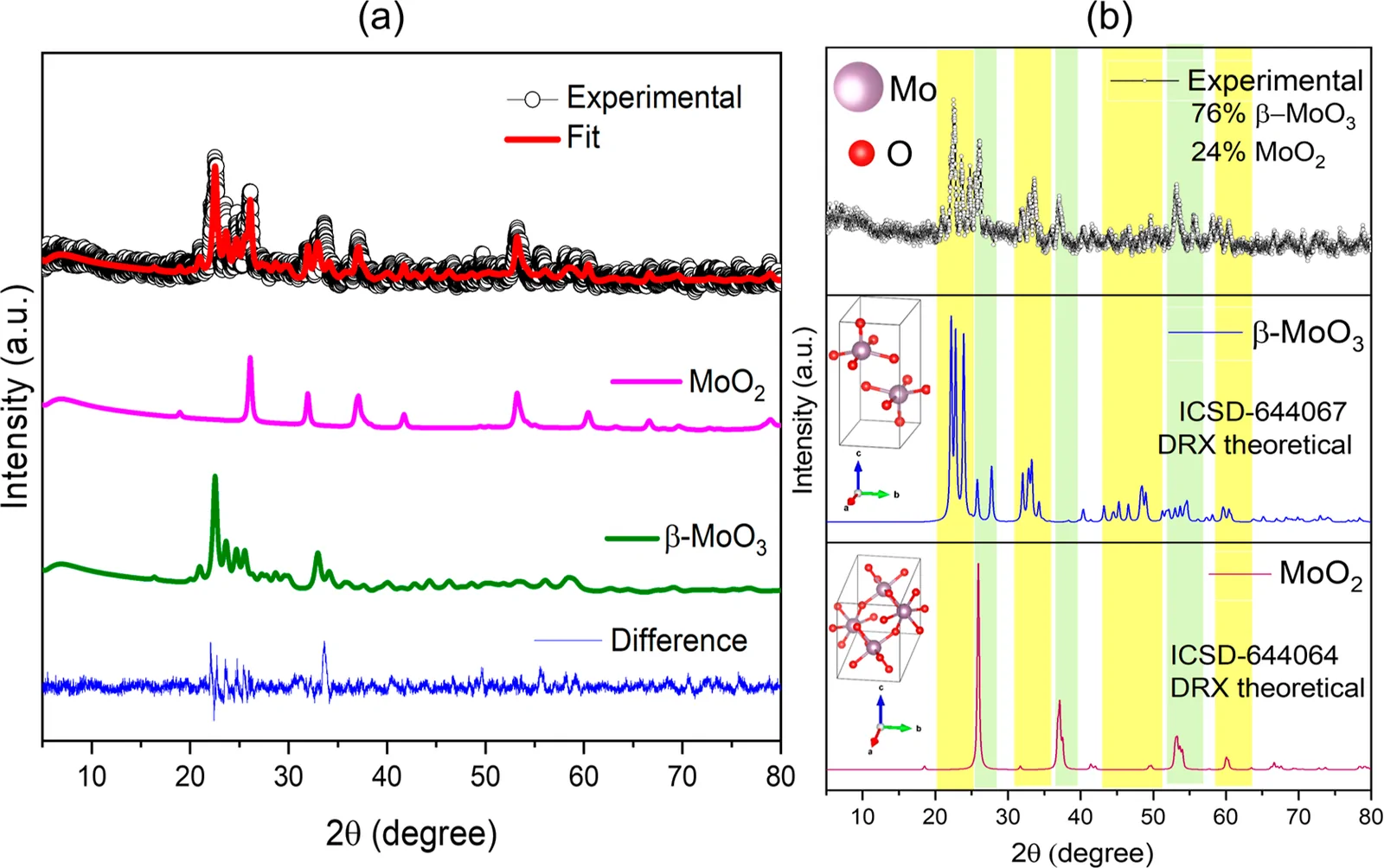
Structural analysis result of the sample thermally treated at 600 °C for 6 h under vacuum: (a) X-ray diffraction pattern evidencing the formation of the β-MoO_3_ phase as the major phase and MoO_2_ as the secondary phase; (b) comparison between the experimental pattern and the theoretical patterns of each crystalline structure.

The observed structural changes are directly associated with the combination of temperature, time, and, most importantly, the employed atmosphere. According to the literature, the α-MoO_3_ phase exhibits thermal stability up to approximately 650 °C under oxidizing atmosphere.^50,51^ Under conventional conditions, the β-MoO_3_ phase is typically formed at temperatures below 200 °C and is gradually converted into the α-MoO_3_ phase at temperatures above 350 °C.^4,5,52^

The structural transition from the β-MoO_3_ phase to the α-MoO_3_ phase involves internal rearrangements of the crystal lattice, including displacements of molybdenum atoms and an increase in the covalent character of Mo–O bonds. This contributes to the stabilization of the thermodynamically favored phase. Under thermal treatment in an oxidizing atmosphere, β-MoO_3_ crystallites tend to grow through coalescence, ultimately leading to the formation of the well-ordered lamellar structure characteristic of the α-MoO_3_ phase.^8^

However, the inverse structural transition (α → β) has not been extensively studied in the literature. Experimental evidence suggests that, under oxygen-deficient atmospheres, the α-MoO_3_ phase may undergo partial reduction to β-MoO_3_ at temperatures around 299 °C. The subsequent reduction of the β-MoO_3_ phase to MoO_2_ may occur via the formation of an intermediate monoclinic β-MoO_2_ phase starting at approximately 450 °C, resulting in the predominant formation of MoO_2_ at temperatures above 600 °C.^53^ Additionally, other studies report that the α-MoO_3_ phase can be directly reduced to MoO_2_ under vacuum atmosphere near 600 °C, without the detectable formation of intermediate phases. These results demonstrate that the structural transformation pathway of the Mo–O system is strongly dependent on the kinetic conditions of the process and the oxygen partial pressure during thermal treatment.^54,55^

## Results of Hyperfine Interactions at ^111^In(^111^Cd) Radioactive Probe

To investigate the phase transition from β-MoO_3_ to MoO_2_ at a local level, time-differential perturbed angular correlation (TDPAC) spectroscopy measurements were conducted as a function of temperature. The nuclear probe ^111^In (^111^Cd) was diffused into α-MoO_3_ microbelts for this purpose. The microbelts were then sealed in evacuated quartz tubes after a small volume of radioactive solution of ^111^InCl_3_ was dropped onto their surface. The amount of ^111^In introduced was extremely low relative to the amount of matrix material and could be considered negligible from a compositional standpoint. Thus, the ^111^In acts as a radioactive probe at the trace level, with no significant influence on the chemical composition or phase stability expected from its presence. The diffusion process took place at 600 °C for 6 h. Figure 6 shows the perturbation functions *R*(*t*) and the corresponding Fast Fourier Transforms (FFT) acquired at 25 °C, 200 °C, 400 °C, 600 °C, and again at 25 °C after completing the thermal cycle. To carry out the TDPAC measurements, the samples were sealed in evacuated quartz tubes (pressure around 10^–3^ mbar).

**Figure 6:**
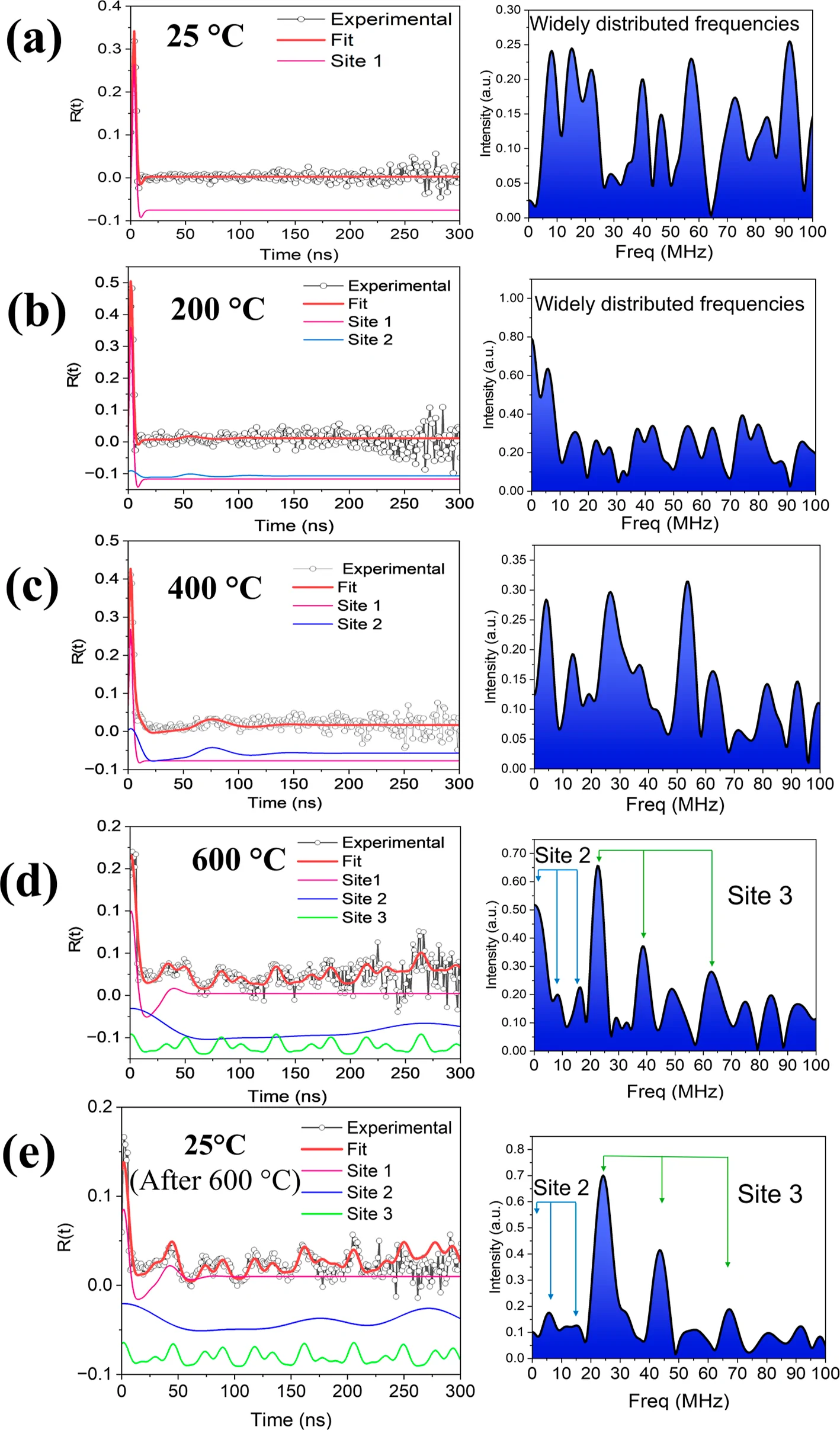
Temporal evolution of *R*(*t*) and the corresponding FFT spectra for the sample under different thermal conditions: (a) 25 °C, (b) 200 °C, (c) 400 °C, (d) 600 °C, and (e) 25 °C after heating up to 600 °C. The left panels show the experimental data, while the right panels display the corresponding FFT spectra, highlighting the distribution of dominant frequencies. In (d) and (e), the arrows indicate the modes associated with sites *S*_2_ and *S*_3_.

The TDPAC spectra obtained are exclusively characterized by electric interactions, evidencing progressive modifications in the hyperfine environment of the ^111^Cd probe as a function of thermal treatment. At the initial room temperature (25 °C), the spectrum is described by a single electric quadrupole interaction associated with site S_1_, characterized by a high quadrupole frequency (ν_*Q*_ ≈ 285 MHz), an asymmetry parameter (η ≈ 0.45), and a relatively broad frequency distribution (δ ≈ 85%). This set of parameters suggests that the probe occupies a highly heterogeneous local environment, typically associated with strongly perturbed regions such as surfaces or grain boundaries, indicating that most probes are not yet incorporated into well-defined crystallographic sites. Due to its highly distributed character, site S_1_ is not considered in the subsequent analysis of hyperfine interactions. Values obtained from the fit of the theoretical function of *R*(*t*) to the experimental data are displayed in Table 1.

**Table 1:** Results from the Fit of Theoretical Function to Experimental Data Showing Values Measured at Indicated Temperature (*T*_M_) for the Hyperfine Parameters: Quadrupole Frequency (ν_*Q*_), the Main Electric Field Gradient Component (*V*_*zz*_), the Asymmetry Parameter (η), and the Site Fraction Population (*f*)

*T*_M_ (°C)	ν_*Q*_ (MHz)	*V*_*zz*_ (V/Å^2^)	η	*f* (%)	observation
200	122	66.0	0.35	3	site S_2_
400	84	45.4	0.30	15	site S_2_
600	24	13.0	0.30	20	site S_2_
600	132	71.4	0.35	11	site S_3_
25	24	13.0	0.30	15	site S_2_
25	149	80.6	0.30	20	site S_3_

At 200 °C (Figure 6b), the emergence of a new hyperfine environment, identified as site S_2_, is observed, albeit with a low relative fraction (∼3%). This site is characterized by a quadrupole frequency (ν_Q_) of 122 MHz, an asymmetry parameter η ≈ 0.35, and a moderate frequency distribution (δ ≈ 15%). The appearance of site S_2_ indicates the onset of local structural rearrangements in the material, with the formation of more defined environments for the nuclear probe, still coexisting with highly disordered regions.

Upon increasing the temperature to 400 °C (Figure 6c), site S_2_ becomes more prominent, with a significant increase in its relative fraction (∼15%). Additionally, a reduction of the quadrupole frequency to 84 MHz is observed, accompanied by an asymmetry parameter η ≈ 0.30 and a decrease in the frequency distribution (δ ≈ 10%). The evolution of these hyperfine parameters evidence a progressive structural reorganization, leading to a more homogeneous and less distributed local environment.

At 600 °C (Figure 6d), the spectra exhibit two well-defined hyperfine environments, identified as sites S_2_ and S_3_. Site S_2_ (with a relative fraction of 20%) presents a quadrupole frequency (ν_*Q*_) of 24 MHz, associated with an asymmetry parameter η ≈ 0.30 and a reduced distribution (δ ≈ 5%), indicating the formation of a relatively homogeneous and stable local environment for the ^111^Cd probe. The presence of this site is clearly evidenced in the corresponding Fourier transform, indicating a dominant hyperfine environment. Simultaneously, site S_3_ emerges with a relative fraction of approximately 11%, characterized by a quadrupole frequency (ν_*Q*_) of 132 MHz, an asymmetry parameter η ≈ 0.35, and a null frequency distribution (δ = 0). These parameters indicate a distinct local environment, more ordered in terms of the electric field gradient (EFG), yet structurally different from site S_2_, suggesting the coexistence of local domains with distinct hyperfine characteristics established at high temperature.

The TDPAC spectrum shown in Figure 6e indicates that, after the thermal treatment (return to room temperature), the structural modifications are irreversible. In this context, sites S_2_ and S_3_ remain well-defined, with hyperfine parameters very similar to those observed at 600 °C. Site S_2_ maintains a quadrupole frequency of ν_*Q*_ ≈ 24 MHz, an asymmetry parameter η ≈ 0.30, and a reduced distribution (δ ≈ 5%), whereas site S_3_ exhibits an increase in the quadrupole frequency to ν_*Q*_ ≈ 149 MHz, accompanied by a reduction of the asymmetry parameter to η ≈ 0.30, while maintaining δ = 0. The persistence of these parameters confirms the stabilization of the hyperfine environments formed at high temperature, reflecting the coexistence of distinct local environments in the material after thermal treatment. Notably, a redistribution of site populations is observed, characterized by a reduction in the fraction of site S_2_ to approximately 15%, accompanied by a significant increase in the fraction of site S_3_ up to about 20%. This behavior suggests a thermally activated transformation in which part of the local environments associated with S_2_ evolve into the more stable configuration represented by S_3_.

After considering previously reported theoretical simulations^56^ for ^111^Cd in α-MoO_3_, we can conclude that our measurements at 600 °C and subsequent measurements at 25 °C do not represent probes at substitutional sites (*V*_*zz*_ = 45.18 V/Å^2^, η = 0.95) or at interstitial sites (*V*_*zz*_ = 54.13 V/Å^2^, η = 0.24) in α-MoO_3_ as reported by Gerami et al.^56^ As *V*_*zz*_ is highly sensitive to the distances between the probe and the charges along the *c*-axis, and considering that the lattice parameters *a, b* and *c* are smaller for MoO_2_ than for β-MoO_3_, we can expect a higher value of *V*_*zz*_ for MoO_2_ compared to β-MoO_3_. This suggests that S_3_ likely represents the ^111^Cd probe at Mo sites in MoO_2_, while S_2_ would be attributed to probes at Mo sites in β-MoO_3_. In addition, comparison of *V*_*zz*_ values with those for other dioxide oxides, such as HfO_2_ (*V*_*zz*_ = 61 V/Å^2^, η = 0.57), with the same space group *P*2_1_/*c* but with different cation coordination,^57^ TiO_2_ and SnO_2_ (*V*_*zz*_ = 56 V/Å^2^, η = 0.12, and *V*_*zz*_ = 48.2 V/Å^2^, η = 0.15, respectively), with similar crystal structure and same cation coordination,^58–60^ and CeO_2_ (*V*_*zz*_ = 53.2 V/Å^2^, η = 0.10) with two oxygen vacancies,^61^ can also reinforce this conclusion.

Furthermore, a recent TDPAC study using the same ^111^In­(^111^Cd) probe reported a quadrupole frequency of approximately 138 MHz attributed to MoO_2_, characterized in the absence of secondary phases.^62^ Although the local symmetry corresponding to this hyperfine environment was not determined, the reported frequency is close to those observed for S_3_ in the present study—132 MHz at 600 °C and 149 MHz after cooling. The agreement between these hyperfine parameters, together with the predominance of MoO_2_ identified by XRD following the TDPAC measurements, supports the interpretation that S_3_ is likely associated with a hyperfine environment related to MoO_2_.

To facilitate a clearer interpretation of the temperature-dependent behavior of the hyperfine parameters, those associated with site S_2_ were initially plotted as a function of temperature, as shown in Figure 7a. This representation allows direct visualization of the individual thermal evolution of each parameter throughout the heating cycle, as well as after cooling. Furthermore, considering the influence of the thermal history of the system, a Kendall correlation matrix for site S_2_ was constructed and is presented in Figure 7b. This matrix enables identification of both the degree of correlation between the hyperfine parameters and temperature, as well as cross-correlations among the parameters themselves. Additionally, in order to investigate similarities among the different thermal states, a hierarchical clustering analysis of the hyperfine parameters of site S_2_ was performed using the agglomerative method with Ward’s criterion, as shown in Figure 7c. This approach allows grouping of the experimental states based on the overall similarity of the hyperfine parameters, providing an integrated view of the structural reorganization induced by the annealing. Given the limited number of thermal conditions investigated, these statistical analyses are interpreted as exploratory tools to identify trends and similarities among the experimental states.

**Figure 7:**
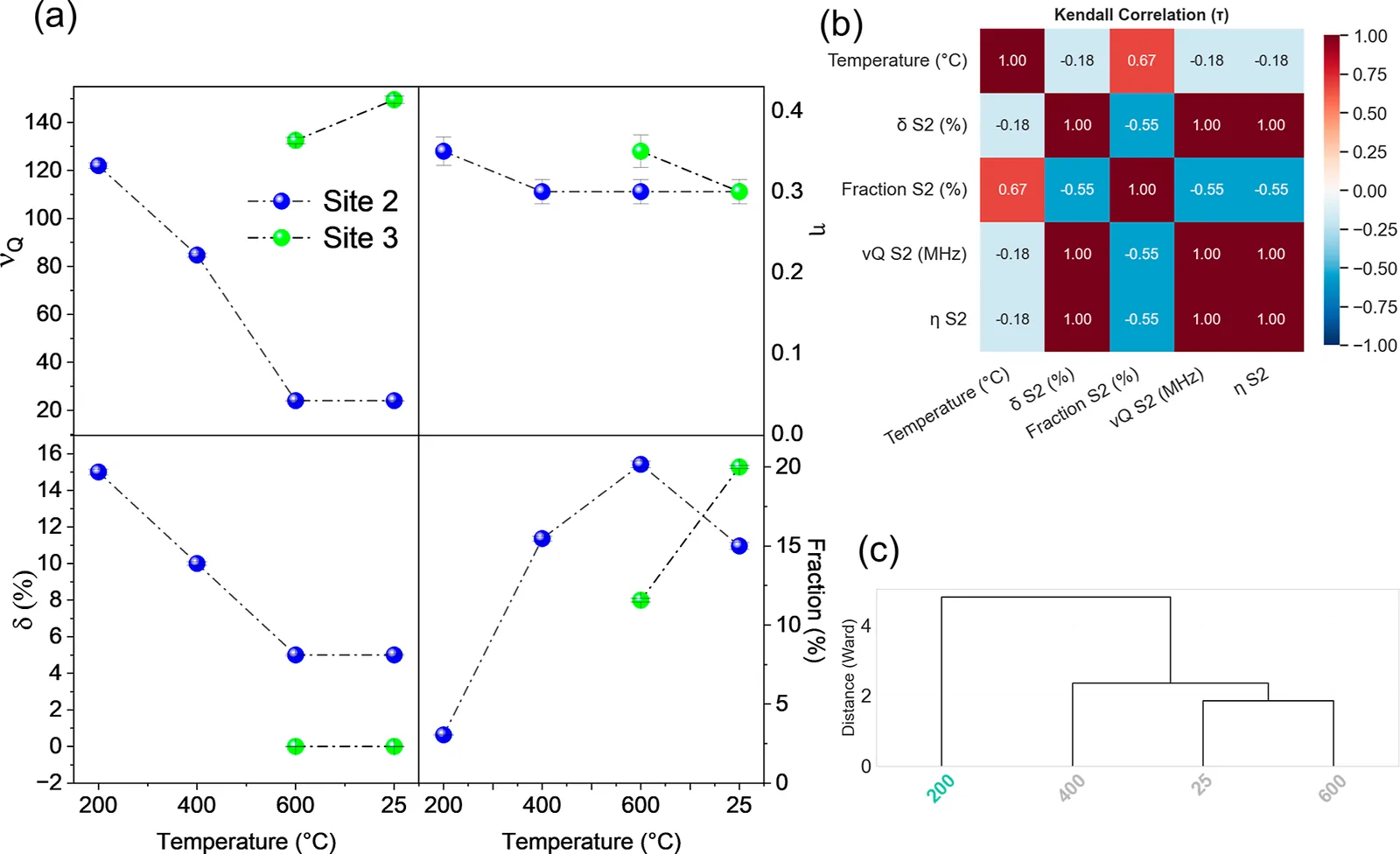
Analysis of hyperfine parameters and statistical correlations as a function of temperature. (a) Evolution of the hyperfine parameters ν_*Q*_, η, δ, and relative fraction for sites *S*_2_ and *S*_3_ as a function of temperature; (b) correlation matrix between temperature and the hyperfine parameters associated with site *S*_2_; and (c) hierarchical clustering dendrogram (Ward’s method) of the thermal conditions, evidencing the formation of two distinct clusters.

The exploratory Kendall correlation matrix suggests systematic trends between temperature and the hyperfine parameters of site S_2_. In particular, the distribution width δ exhibits a negative correlation with temperature, indicating that, as the temperature increases, the distribution becomes narrower. This behavior reflects a progressive reduction in local disorder, suggesting that the probe evolves toward a more well-defined hyperfine environment during the thermal treatment. Similarly, the asymmetry parameter η also shows a negative correlation with temperature, indicating that the electric field gradient becomes more symmetric at higher temperatures, consistent with a reduction in structural distortion.

In contrast, the fraction associated with site S_2_ presents a positive correlation with temperature. This trend is mainly driven by the significant increase in the site population between 200 °C and higher temperatures (400 and 600 °C), where the occupation of this site becomes substantially more pronounced. Therefore, the correlation reflects not only a monotonic trend with temperature, but also the strong weight of the population change across the thermal evolution.

The hierarchical clustering analysis (Figure 7c) identifies two distinct groups. The condition at 200 °C forms an isolated cluster, while the conditions at 400 °C, 600 °C, and 25 °C (after cooling) are grouped into a second cluster. This result indicates that the hyperfine environment at 200 °C is significantly different from those observed at higher temperatures and after cooling. In contrast, the clustering of 400 °C, 600 °C, and 25 °C suggests that the structural configuration achieved at high temperature is largely preserved upon cooling, evidencing the stabilization of the hyperfine environment of site S_2_.

The behavior observed for the hyperfine parameters in Figure 7a also suggests the progressive formation of oxygen vacancies during the thermal treatment under vacuum. Under oxygen-deficient conditions, the stabilization of the metastable β-MoO_3_ phase is frequently associated with the generation of locally oxygen-deficient environments, which promote partial reduction of Mo^6+^ species and induce local lattice distortions. The evolution of site S_2_, particularly the decrease in both ν_*Q*_ and δ with increasing temperature, can be interpreted as evidence of a progressive reorganization of the local coordination environment around the probe nuclei driven by oxygen vacancy formation. Such defects are expected to strongly modify the local electric field gradient through changes in charge distribution, Mo–O bond covalency, and local symmetry as reported in ref 61 for Cd doping in CeO_2_. Therefore, the stabilization of β-MoO_3_ at 600 °C under vacuum is not solely a consequence of the reducing atmosphere, but is intrinsically connected to the generation and redistribution of oxygen vacancies, which act as structural defects that favor the persistence of this metastable phase prior to the irreversible transformation into MoO_2_.

### Structural and Morphological Characterization after TDPAC Measurements

Within the context of the evidenced phase transition, although it is not possible to determine the symmetry associated with the frequencies obtained for the S_2_ and S_3_ sites after treatment at 600 °C, the results suggest that they can be attributed to MoO_2_-related environments, considering that the phase transition from β-MoO_3_ to MoO_2_ under vacuum at temperatures on the order of 600 °C is predicted in the literature.^53^ This hypothesis becomes even more plausible in light of the results obtained for the sample after the TDPAC measurements, as presented in Figure 8. Figure 8a–f show the SEM results and elemental analysis, indicating the absence of a predominant morphology, which suggests coalescence of the microbelts as a consequence of the thermal treatment under vacuum. The EDS analysis confirms the presence of Mo and O after thermal treatment. Figure 8g illustrates the structural changes that occurred during the TDPAC measurements: the XRD pattern indicates monoclinic MoO_2_ (ICSD-644064, *P*2_1_/*c*) as the major phase (90%) and β-MoO_3_ (ICSD-644067) as the minor phase (10%), according to the refinement performed using the Rietveld method.

**Figure 8:**
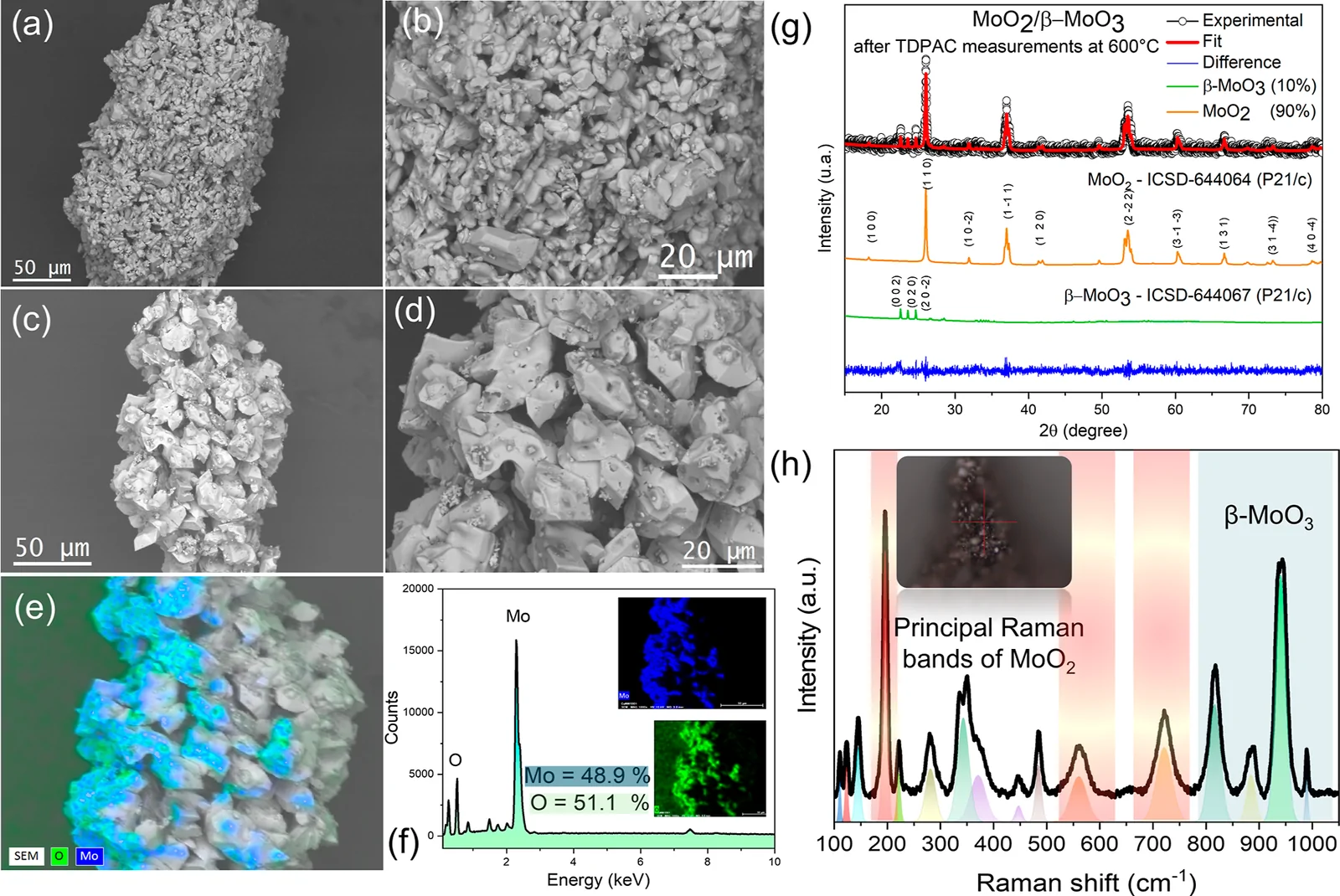
Results of XRD, SEM/EDS, and Raman analyses of the sample treated at 600 °C for 6 h after the TDPAC measurements: (a–d) scanning electron microscopy (SEM) micrographs at different magnifications, evidencing the ill-defined morphology of the sample (a and c) and surface regions of the sample (b and d); (e) EDS analysis region; (f) EDS spectrum; (g) X-ray diffraction pattern; (h) Raman spectrum of the sample, highlighting the characteristic bands of MoO_2_ and β-MoO_3_.

Figure 8h shows the result of the Raman spectroscopy analysis performed after the TDPAC measurements. The spectrum exhibits bands at approximately 111, 123, 145, 196, 222, 281, 343, 371, 485, 561, 722, 817, 885, 941, and 990 cm^–1^, indicating the coexistence of different structural environments and oxidation states of molybdenum.^7,63,64^ It is observed that the band at approximately 196 cm^–1^ is the most intense feature in the spectrum, a behavior characteristic of the monoclinic MoO_2_ phase. This vibrational mode is attributed to collective vibrations of the crystal lattice, mainly involving displacements and rotations of distorted MoO_6_ octahedra, which are strongly coupled through Mo–O–Mo bonds. The high intensity of this band is commonly associated with the metallic character of MoO_2_, in which low-frequency modes exhibit strong variations in electronic polarizability, resulting in enhanced Raman intensities.^47,63^ The additional bands observed at approximately 222, 281, 343, and 371 cm^–1^ are also typical of MoO_2_ and correspond to other vibrational modes associated with internal deformations and collective motions of the distorted MoO_6_ octahedra. In the intermediate frequency region, the bands located at approximately 485 and 561 cm^–1^ are attributed to vibrations involving stretching and angular deformations of the Mo–O–Mo bonds, while the band at approximately 722 cm^–1^ is widely reported as one of the most representative vibrational modes of the MoO_2_ phase.^7,47,63,65^

On the other hand, the bands observed in the high-frequency region, at approximately 817, 885, 941, and 990 cm^–1^, are not attributed to MoO_2_. These modes are consistent with the presence of residues of the β-MoO_3_ phase, which crystallizes in a metastable monoclinic structure and exhibits a Raman band pattern distinct from that of the α-MoO_3_ phase.^64^ In particular, the band around approximately 990 cm^–1^ is assigned to the terminal stretching mode ν­(Mo=O), characteristic of Mo^6+^ species, while the bands in the 880–950 cm^–1^ range are associated with distorted internal modes of the MoO_6_ octahedra, which are typical of β-MoO_3_ and absent in stoichiometric MoO_2_.^64,65^ The relatively lower intensity of these high-frequency bands, when compared to the dominant band at approximately 196 cm^–1^, indicates that β-MoO_3_ is present only as a residual phase, in full agreement with the X-ray diffraction results, which identify MoO_2_ as the major phase of the analyzed material.

Thus, the integrated analysis of TDPAC, XRD, SEM/EDS, and Raman spectroscopy provides a consistent and complementary picture of the structural evolution of the material, evidencing the irreversible transformation into a predominantly MoO_2_ phase, with the coexistence of residual β-MoO_3_ domains.

## Conclusion

This study establishes a comprehensive understanding of the formation, transformation, and stabilization of MoO_*x*_ phases derived from a chelated molybdenum precursor. Although the initial synthetic route, the thermal decomposition of molybdenum­(II) acetate, yields an amorphous chelated material rather than directly crystalline nanoparticles, it provides a versatile platform for controlled phase engineering through postsynthesis thermal treatments.

A key finding is the decisive role of the annealing atmosphere in governing both structural and morphological evolution. Under oxidizing conditions, α-MoO_3_ remains stable while exhibiting significant morphology modulation from microbelts to nanosheets, demonstrating that morphology can be tuned independently of crystal phase. In contrast, vacuum conditions promote reduction pathways, enabling the formation of β-MoO_3_ and its subsequent transformation into MoO_2_.

Most importantly, the β-MoO_3_ → MoO_2_ transition is resolved at the atomic scale through TDPAC measurements, revealing a progressive ordering process culminating in a stable MoO_2_ local environment above 600 °C. The identification of distinct hyperfine sites, supported by statistical correlation and clustering analysis, provides direct evidence of an irreversible phase transformation governed by oxygen chemical potential. The excellent agreement between hyperfine data and postannealing XRD, SEM, and Raman results confirms the robustness of this multiscale approach.

Overall, this work demonstrates that the combination of controlled thermal processing and local probe spectroscopy enables precise tuning of phase composition, morphology, and local structure in MoO_*x*_ systems. These insights are highly relevant for applications where phase-dependent properties are critical, such as catalysis, sensing, and energy conversion, and position TDPAC as a powerful technique for guiding the design of functional oxide materials.

## Experimental Section

With the aim of synthesizing nano- and microstructured molybdenum oxides via the thermal decomposition method, molybdenum­(II) acetate (Merck, 98%) was used as the organometallic precursor, while oleylamine (Merck, 70%) and commercial palm kernel oil were employed as the surfactant and binding agents, respectively.

In this context, 150 mg of the precursor were added to a three-neck round-bottom flask containing oleylamine and palm kernel oil in a 1:1 volume ratio. The mixture was homogenized in an ultrasonic bath and subsequently heated under reflux in a nitrogen atmosphere at a heating rate of 10 °C min^–1^. The process was carried out in two stages: a nucleation step at 240 °C for 10 min, followed by a growth stage at 340 °C for 3 h.

After the synthesis, a molybdenum-based chelate was obtained and initially characterized by X-ray diffraction (XRD), scanning electron microscopy (SEM), and Fourier-transform infrared spectroscopy (FTIR). Subsequently, the chelate was subjected to two thermal treatments at the same temperature but for different durations, in order to evaluate the influence of time on the crystallization process of molybdenum oxide phases (MoO_*x*_). The thermal treatments were carried out at 700 °C for 10 min and at 700 °C for 3 h, respectively.^40^ The samples obtained after the thermal treatments were characterized by XRD and SEM. In addition, the sample treated for 3 h was analyzed by Raman spectroscopy. Under this thermal treatment condition, the formation of α-MoO_3_ microbelts was evidenced.

In order to investigate the influence of temperature and annealing atmosphere on the structural characteristics of the microbelts (formed after annealing for 3 h under ambient atmosphere), the samples were divided into two groups and subjected to different annealing conditions. One set was treated at 800 °C in an ambient, oxygen-rich atmosphere, while the other was subjected to thermal treatment at 600 °C under vacuum, with an annealing time of 6 h for both conditions. The choice of these conditions is based on previous studies suggesting that thermal treatments in oxidizing atmospheres favor the stabilization of the α-MoO_3_ phase and promote the growth of lamellar nanostructures, whereas reducing or low-oxygen atmospheres, such as vacuum, induce the formation of oxygen defects, partial reduction of the molybdenum oxidation state, and possible structural transformations into substoichiometric phases, such as MoO_2_, at temperatures around 600 °C; however, these processes are still under investigation.^48,49,53^ After annealing, the samples were subjected to structural characterization by X-ray diffraction (XRD) and scanning electron microscopy (SEM).

### Radioactive Sample Preparation

With the aim of performing a more comprehensive local investigation of the possible phase transitions reported for α-MoO_3_ up to 600 °C under vacuum conditions,^53^ radioactive samples corresponding to this set were prepared, enabling their characterization by means of the time-differential perturbed angular correlation (TDPAC) technique. Because the main objective of this work was to observe the phase transformations predicted in the literature for the MoO_*x*_ system under low-oxygen conditions, with particular interest in the evolution of hyperfine environments associated with the transformation from β-MoO_3_ to MoO_2_, we used TDPAC measurements only on the vacuum-annealed samples. In addition, because the hyperfine characterization of α-MoO_3_ using the ^111^Cd probe has already been reported in the literature, it was not the primary focus of this study.

TDPAC measurements allow one to obtain the anisotropy ratio function as a function of time, expressed as $R\left(t\right)=A_{22}\sum_{i}f_{i}G_{22}^{i}\left(t\right)$ , where *f*_*i*_ corresponds to the fraction of probe nuclei located in different crystallographic environments and $G_{22}^{i}\left(t\right)$ represents the perturbation function associated with each site. For the ^111^In­(^111^Cd) system, the angular correlation coefficient *A*_22_ has a value of −0.18, which requires the representation of the *R*(*t*) function with inversion of the vertical axis. Additional information on the fundamentals and applications of the TDPAC technique can be found in the specialized literature.^35^

In the specific case of electric hyperfine interactions of the quadrupole type, the perturbation factor can be described by $G_{22}\left(t\right)=\sum_{n}S_{kn}\cos\left(n\omega_{0}t\right)$ , where the fundamental frequency ω_0_ is given by ω_0_ = *g*_*n*_(η)­ν_*Q*_, with the quadrupole frequency defined as ν_*Q*_ = *eQV*_*zz*_/*h*. Both the frequency ω_0_ and the coefficients *S*_*kn*_ depend on the asymmetry parameter η, defined as η = (*V*_*xx*_ – *V*_*yy*_)/*V*_*zz*_. From TDPAC measurements, it is possible to determine the values of η and ν_*Q*_, by fitting theoretical functions to the experimental data^36^ allowing the calculation of the components *V*_*cc*_ (*c* = *x*, *y*, *z*) of the electric field gradient tensor. The parameter η indicates the degree of deviation from axial symmetry of the local charge distribution, while the component *V*_*zz*_ provides information on the electronic density in the vicinity of the probe nuclei. Within the scope of the TDPAC measurements, the probe (or radioactive tracer) ^111^Cd was employed due to its favorable nuclear properties. This nucleus is generated from the electron-capture decay of the parent nucleus ^111^In, resulting in the formation of ^111^Cd.

In order to promote the incorporation of the nuclear probe into the crystalline environment, a pellet was prepared from α-MoO_3_ microbelts obtained by annealing the precursor chelate at 700 °C for 3 h under ambient atmosphere. The powdered material was compacted by uniaxial pressing in a hydraulic press under a load of 4 tons, resulting in a mechanically stable pellet. After pellet preparation, a few drops of an aqueous ^111^InCl_3_ solution, with an approximate volume of 20 μL, were deposited onto its surface. The pellet was then vacuum-dried in a desiccator in order to remove residual moisture. Subsequently, a second vacuum-drying step was carried out in a quartz tube for approximately 1 h, after which the tube was sealed under vacuum. The sample was then subjected to a thermal treatment at 600 °C for 6 h, aiming to promote the effective diffusion of the probe nuclei into the bulk of the material.

After incorporation of the radioactive probe, TDPAC measurements were performed as a function of temperature. The spectra were acquired at 25 °C, 200 °C, 400 °C, 600 °C, and again at 25 °C. This final room-temperature measurement was intended to investigate the reversibility of possible structural transitions induced by the thermal treatment. It is emphasized that the acquisition time for each spectrum was approximately 6 h, during which the perturbed anisotropic correlation functions *R*(*t*) were recorded. After completion of the TDPAC measurements and the adequate decay of the radioactive activity, the sample was subjected to further structural and morphological characterizations by X-ray diffraction (XRD), scanning electron microscopy (SEM), and Raman spectroscopy.

The TDPAC measurements were carried out at the Hyperfine Interactions Laboratory (LIH), located at the Research Reactor Center (CERPq/IPEN). Structural characterizations by X-ray diffraction (XRD) were performed using a SmartLab diffractometer equipped with a high-resolution two-dimensional detector (HPD 2D), operating with Cu Kα radiation (λ = 1.54056 Å), an accelerating voltage of 40 kV, and a current of 40 mA. The diffraction patterns were collected over the angular range from 10° to 80° in 2θ, with an angular step of 0.01° and a scanning speed of 1° min^–1^. Morphological characterization was carried out by field-emission scanning electron microscopy (FEG–SEM) using a JSM-6701F microscope (JEOL), coupled to an energy-dispersive spectroscopy (EDS) detector. Raman spectroscopy measurements were performed using an XploRA Plus micro-Raman system (Horiba), with a 532 nm excitation laser, covering the spectral range from 100 to 1000 cm^–1^. Figure 9 provides a schematic overview of the methodology applied in sample preparation and of the characterizations performed for each group of samples (radioactive and nonradioactive) described in this section.

**Figure 9:**
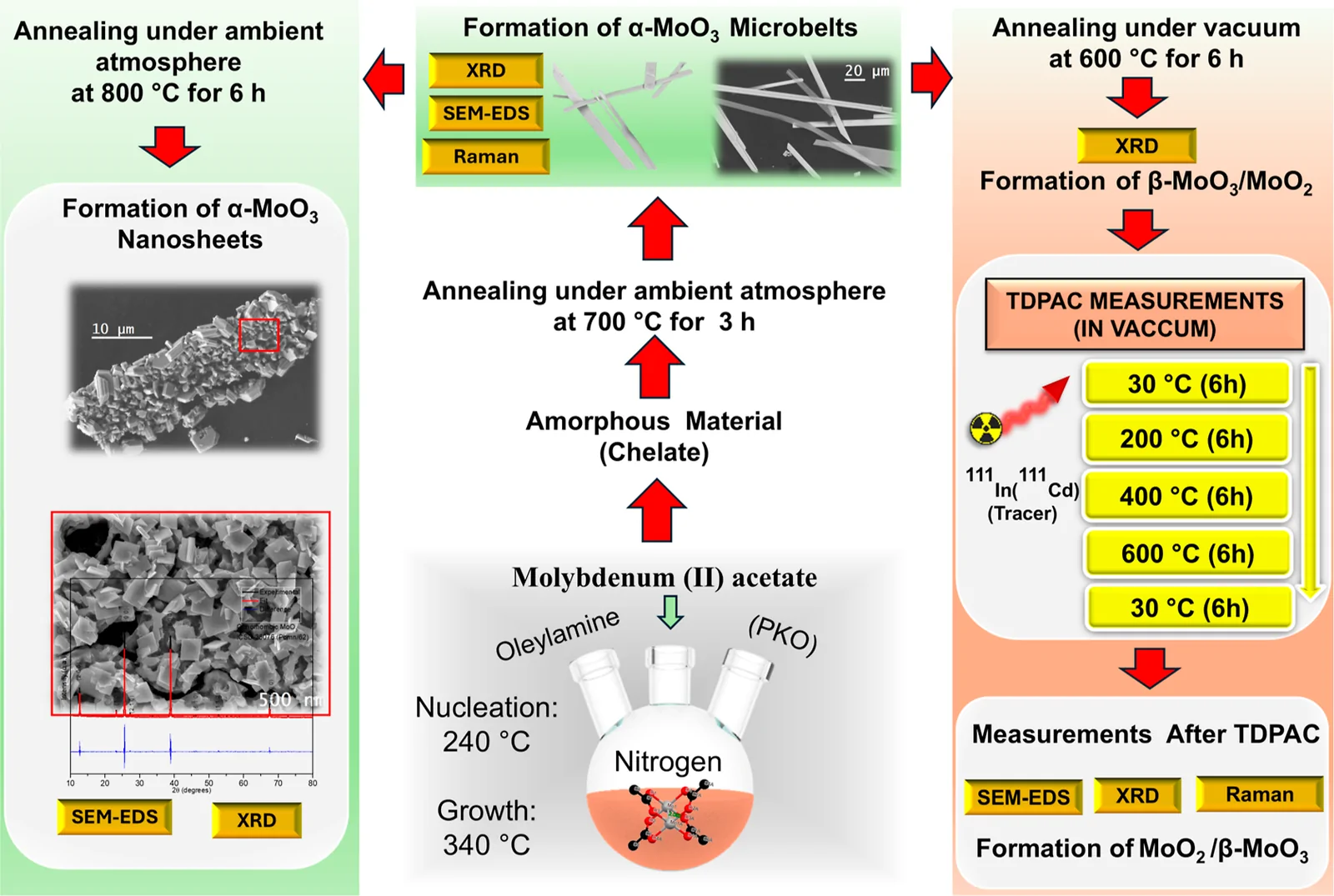
Schematic representation of the methods applied for the synthesis of molybdenum oxide nano- and microstructures, indicating the characterizations performed on radioactive and nonradioactive samples. At the center, the preparation of the α-MoO_3_ sample and its corresponding characterization techniques. On the left, thermal treatment under an oxidizing atmosphere and the associated characterizations; on the right, thermal treatment under vacuum and the techniques applied to radioactive samples.

## Supplementary Material



## Notes

X-ray diffraction pattern and scanning electron microscopy of molybdenum­(II) acetate after thermal treatment at 700 °C for 10 min (PDF)
