# Supplementary material for: Atmosphere-Driven Phase Engineering in MoO x : From α‑MoO3 Microbelts to MoO2 Revealed by Atomic-Scale Hyperfine Probes
Source: ACS Mater Au. 2026 Oct 15;6(6):1513–26. doi: 10.1021/acsmaterialsau.6c00153 (PMC13760631; doi:10.1021/acsmaterialsau.6c00153)
Supplement: Supplementary file 1 [file mg6c00153_si_001.pdf]

Atmosphere-Driven Phase Engineering in MoOx: From α-MoO_3 Microbelts to MoO_2 Revealed by Atomic-Scale Hyperfine Probes

Katiusse Soares de Souza,[†] Crystian W. C. Silva,[†] Gabriel A. Cabrera-Pasca,[‡] Juliana Schell,[¶,§] and Artur Wilson Carbonari[*,†,¶]

†*Instituto de Pesquisas Energéticas e Nucleares, IPEN-CNEN/SP, 05508-000, São Paulo, SP, Brazil*

‡*Universidade Federal do Pará (UFPA), Abaetetuba – Pará, 09210-580, Brazil*

¶*European Organization for Nuclear Research (CERN), CH-1211, Geneva, Switzerland*

§*Institute for Materials Science and Center for Nanointegration Duisburg-Essen (CENIDE), University of Duisburg-Essen, 45141, Essen, Germany*

E-mail: carbonar@ipen.br

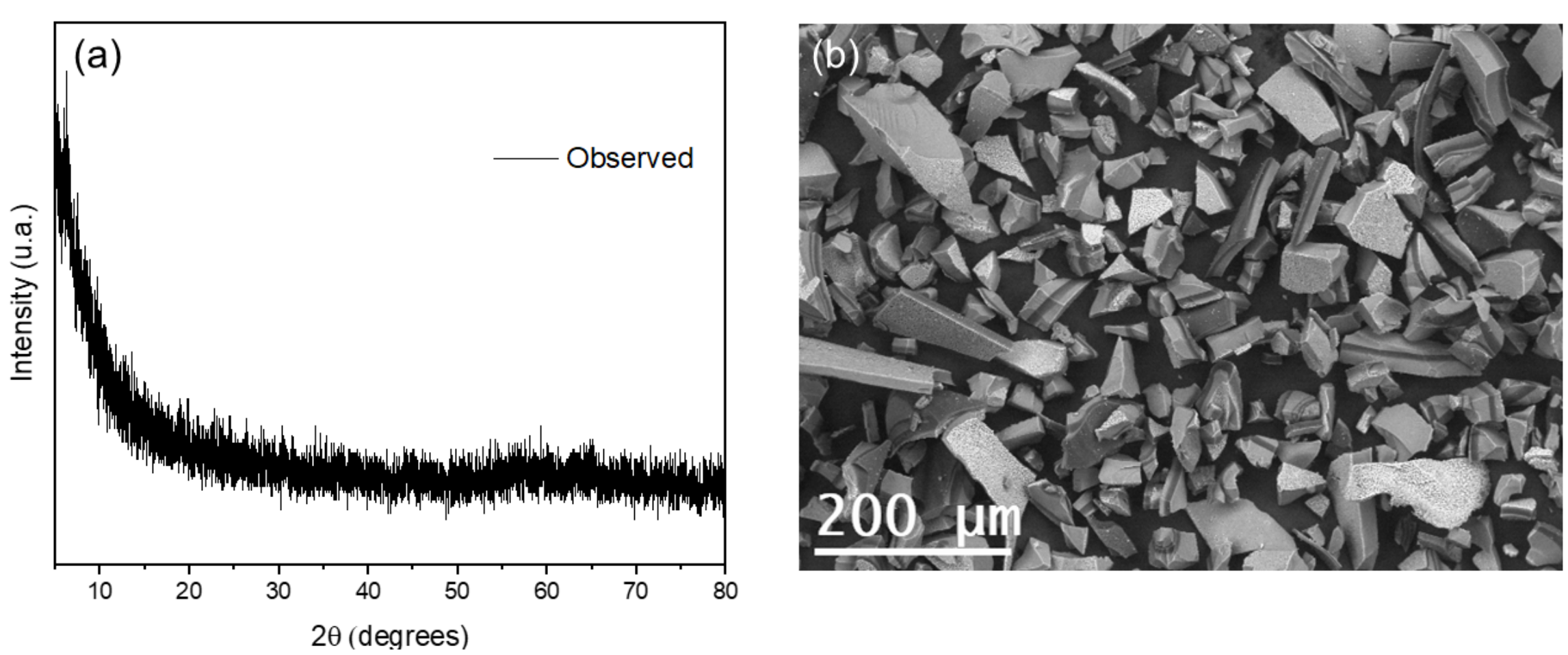

Figure S1: Chelate obtained from molybdenum(II) acetate and thermally treated at 700 °C for 10 min. (a) XRD pattern; (b) SEM micrograph showing the absence of a well-defined morphology.
